# Acute Exercise Improves Motor Memory Consolidation in Preadolescent Children

**DOI:** 10.3389/fnhum.2017.00182

**Published:** 2017-04-20

**Authors:** Jesper Lundbye-Jensen, Kasper Skriver, Jens B. Nielsen, Marc Roig

**Affiliations:** ^1^Department of Nutrition, Exercise and Sports, University of CopenhagenCopenhagen, Denmark; ^2^Department of Neuroscience and Pharmacology, University of CopenhagenCopenhagen, Denmark; ^3^Copenhagen Centre for Team Sport and Health, University of CopenhagenCopenhagen, Denmark; ^4^Memory and Motor Rehabilitation Laboratory (MEMORY-LAB), Feil and Oberfeld Research Centre, Jewish Rehabilitation Hospital, Montreal Center for Interdisciplinary Research in Rehabilitation (CRIR)Laval, QC, Canada; ^5^School of Physical and Occupational Therapy, Faculty of Medicine, McGill UniversityMontreal, QC, Canada

**Keywords:** motor memory, consolidation, retention, exercise, learning, children

## Abstract

**Objective**: The ability to acquire new motor skills is essential both during childhood and later in life. Recent studies have demonstrated that an acute bout of exercise can improve motor memory consolidation in adults. The objective of the present study was to investigate whether acute exercise protocols following motor skill practice in a school setting can also improve long-term retention of motor memory in preadolescent children.

**Methods**: Seventy-seven pre-adolescent children (age 10.5 ± 0.75 (SD)) participated in the study. Prior to the main experiment age, BMI, fitness status and general physical activity level was assessed in all children and they were then randomly allocated to three groups. All children practiced a visuomotor tracking task followed by 20 min of rest (CON), high intensity intermittent floorball (FLB) or running (RUN) with comparable exercise intensity and duration for exercise groups. Delayed retention of motor memory was assessed 1 h, 24 h and 7 days after motor skill acquisition.

**Results**: During skill acquisition, motor performance improved significantly to the immediate retention test with no differences between groups. One hour following skill acquisition, motor performance decreased significantly for RUN. Twenty-four hours following skill acquisition there was a tendency towards improved performance for FLB but no significant effects. Seven days after motor practice however, both FLB and RUN performed better when compared to their immediate retention test indicating significant offline gains. This effect was not observed for CON. In contrast, 7 days after motor practice, retention of motor memory was significantly better for FLB and RUN compared to CON. No differences were observed when comparing FLB and RUN.

**Conclusions**: Acute intense intermittent exercise performed immediately after motor skill acquisition facilitates long-term motor memory in pre-adolescent children, presumably by promoting memory consolidation. The results also demonstrate that the effects can be accomplished in a school setting. The positive effect of both a team game (i.e., FLB) and running indicates that the observed memory improvements are determined to a larger extent by physiological factors rather than the types of movements performed during the exercise protocol.

## Introduction

During the recent years, there has been an increasing focus on the potential effects of exercise on health, cognitive functions and also learning. The quality of public education has rarely been more debated than it is currently, with the discussion focusing on measures that can optimize academic performance of school children (Danish Ministry of Education, 2014). One of the means by which authorities are presently looking to facilitate academic performance in school children, is by incorporating more physical activity in and outside physical education (PE; Alvang, 2010). This initiative is supported by research indicating that there is a positive relation between high levels of aerobic fitness (Åberg et al., 2009; Lambourne et al., 2013), participation in vigorous physical activity (Coe et al., 2006) and academic achievements, even when time is taken from the normal curriculum and dedicated to exercise (Sallis et al., 1999; Ahamed et al., 2007).

These findings are part of a larger body of research showing that exercise can be beneficial for a variety of brain functions (for reviews see Hillman et al., 2008; Taubert et al., 2015). Both chronic and acute exercise can have beneficial effects on cognitive functions (Hillman et al., 2009, 2014) and facilitate the formation and retention of several types of memory (Roig et al., 2013). In children, chronic exercise is associated with significant memory-related benefits such as better academic performance (Coe et al., 2006; Singh et al., 2012; Booth et al., 2014) and cognitive functions (Sibley and Etnier, 2003; Hillman et al., 2014). Similarly, studies investigating the effects of an acute bout of exercise on memory have documented positive effects on cognitive functions including memory in adults (Winter et al., 2007; Kamijo et al., 2009; Labban and Etnier, 2011) and children (Hillman et al., 2009; Pesce et al., 2009; Ellemberg and St-Louis-Deschênes, 2010). Additionally, a meta-analysis found acute exercise to have small but consistent effects on simultaneous or subsequent performance of a memory-related task (Chang et al., 2012). While both chronic and acute exercise can benefit memory functions and effects may be interrelated, the distinction is important since the underlying mechanisms may differ and effects may relate to different aspects of memory.

Memory can crudely be divided into declarative and nondeclarative memory, and a major emphasis is placed on formation and retention of declarative memory in the educational system, e.g., geographical knowledge or recall of names (Squire, 1992). Declarative memory has also been a main focus for the majority of studies mentioned in the previous paragraph. Acquisition and retention of skills does however also play a major role both in the educational curriculum and in the lives of children and adults in general and skill learning requires the formation and retention of procedural or motor memory. It is thus also relevant to investigate how exercise may influence motor memory functions.

Recent studies have demonstrated that motor skills in preadolescent children are related to objective measures of cognitive functions, academic performance (Geertsen et al., 2016) and to academic achievement in adolescence (Kantomaa et al., 2013), and that daily PE and increased focus on motor skill training during compulsory school years can improve both motor skills and academic performance in adolescence (Ericsson and Karlsson, 2014). Finally, since motor (or procedural) memory is both associated with motor skills, but also supports language (Ullman, 2004) and certain social skills (Lieberman, 2000), motor skills may additionally subserve academic learning. Since motor skills are important in everyday life, and we need to acquire and retain a multitude of skills throughout life, these findings justify an increased focus on the principles and mechanisms involved in motor skill learning and motor memory in children.

Considering the effects of acute exercise on skill learning and motor memory, Roig et al. (2012) demonstrated in adults that an acute bout of intense exercise can facilitate long-term memory of a novel motor skill, when performed either before or after initial motor practice (Roig et al., 2012). The study also revealed that the effect was larger when exercise was performed after learning, indicating that exercise benefits the consolidation processes subserving retention of motor memory. Recently, it has been demonstrated also in adults, that consolidation and motor memory is influenced by the timing and intensity of exercise following skill learning (Thomas et al., 2016a, c). Thus, an acute bout of high intensity exercise performed in close temporal proximity to the skill acquisition and thus in the early consolidation phase is preferable for influencing motor memory consolidation positively. However, since these studies were conducted in a lab setting with able-bodied adult male subjects, it remains unknown whether acute exercise can benefit motor memory consolidation in preadolescent children. This question is important to elucidate given the potential for promoting skill learning in children, and in order to elucidate neuroplastic mechanisms underlying memory functions in children. Furthermore, it remains unknown whether the type of the employed exercise protocol plays a role for eliciting positive effects of exercise on motor memory.

The aim of this study was therefore to investigate if a single bout of intense exercise performed after motor skill learning, promotes long-term motor memory in preadolescent children since this has not been investigated in previous studies. Our main hypothesis was that long-term motor memory would be improved in children performing an intense physical activity after skill acquisition, compared to a passive control group. Additionally, we wanted to elucidate whether different types of intense exercise may affect long-term memory differentially.

## Materials and Methods

### Participating Children

Seventy-eight ethnically diverse children from 3rd and 4th grade were recruited to participate in the study (see Table 1). They were all naïve to the visuomotor accuracy-tracking task (MT) used to assess skill learning and retention of motor memory. Exclusion criteria for participation were: history of neurological or psychiatric diseases as well as current intake of medications affecting the central nervous system. The legal guardians of all participating children gave informed written consent on behalf of their child prior to participation. One child withdrew consent before completing the experiment. Blocked-randomization was used to assign children to either running (RUN), floorball (FLB) or control (CON) groups. The groups were matched on gender, age, BMI and fitness level since these factors may influence the effect of acute exercise on performance in cognitive tests, and possibly motor performance (Kamijo et al., 2009; Stroth et al., 2009). The study was performed in accordance with the Declaration of Helsinki II. The ethics committee for the Greater Copenhagen area approved the study (protocol: H-2-2012-169).

**Table 1:** **Characteristics of the children in the control (CON), floorball (FLB) and running (RUN) groups**.

	CON	FLB	RUN
Participants (*n*)	26	26	25
Boys/Girls	13/13	16/10	15/10
Age (Years)	10.3 ± 0.6	10.4 ± 0.7	10.8 ± 0.8*
BMI (Weight/Height^2^)	18.3 ± 2.6	17.1 ± 2.0	17.7 ± 2.9
Cardiovascular fitness (VO_2peak_ mlO2/kg/min)	42.4 ± 3.4	42.5 ± 3.6	41.1 ± 1.9
Physical Activity (PAQ-C, METS)	3.43 ± 0.6	3.41 ± 0.5	3.3 ± 0.7
HR_avg_ during exercise	NA	197 ± 7^+^	191 ± 8
HR_peak_ during exercise	NA	199 ± 6	201 ± 10

Data are reported as mean ± SD. BMI = Body mass index, Cardiovascular fitness is estimated peak VO_2_ consumption using the yoyo-test, PAQ-C, General Physical activity level questionnaire for children; METs, Metabolic Equivalents; HR, Heart rate). *Denotes significantly different from other groups (*p* < 0.05). ^+^Denotes significantly different from RUN (*p* < 0.05).

### Study Design

#### Design Overview

**Figure 1:**
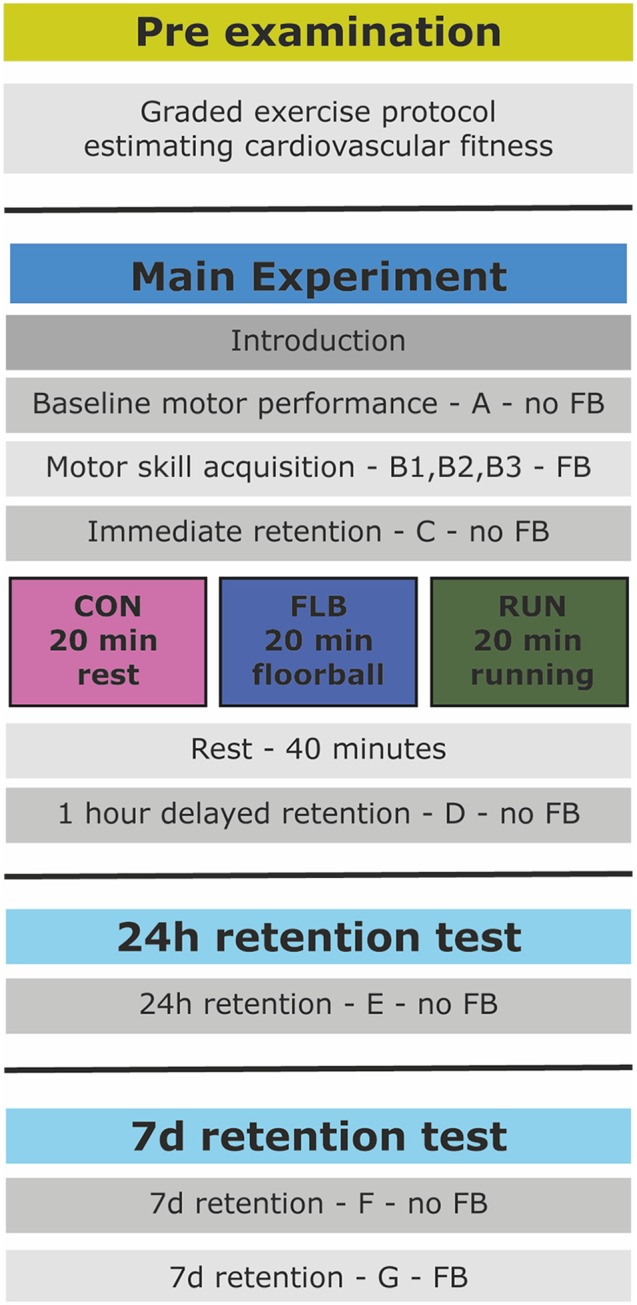
**Schematic overview of the experimental design**. FB, Augmented Feedback; CON, control group; FLB, floorball group; RUN, running group.

The experiment was designed as a randomized controlled trial and included four separate sessions (Figure 1). The pre-examination consisted of an aerobic fitness test to assess fitness level. The following session was the main experiment during which the children practiced the motor task. After motor practice the children rested (CON), played FLB or ran (RUN) for 20 min. Retention tests were performed 1 h, 24 h and 7 days after initial practice of the motor task.

#### Pre-Examination

At least 1 week prior to the main experiment, all children completed a Yo-Yo Interval Restitution Children’s test to assess their aerobic fitness. This test provides an estimate of the maximal oxygen consumption (VO_2-peak_; Bendiksen et al., 2013). During the test, heart rate was monitored (Polar Team System, Kempele, Finland).

#### Main Experiment and Retention Tests

Children were tested in groups of four, with all four being assigned to the same experimental group. They were seated at independent workstations along with an experimenter that followed them throughout the experiment. Initially children were given a brief description of the experiment. Afterwards, the weight, height and body composition (Innerscan, Tanita, Tokyo, Japan) of each child was measured. Additionally, the children completed an Edinburgh Handedness Questionnaire to assess handedness and Physical Activity Questionnaire for Children (PAQ_C_) to estimate daily physical activity level (Kowalski et al., 2004).

Following this, the children practiced MT (see detailed information below). Immediately after motor practice, the children engaged in 20 min of rest (CON), FLB or running (RUN). After the interventions, all children rested 40 min during which they were allowed to read magazines or watch cartoons.

The motor task employed in this study was a modified version of the visuomotor accuracy tracking task previously applied in other experiments (Roig et al., 2012; Thomas et al., 2016a,b,c). In short, the children were comfortably seated at a table, with their forearm resting on the table. Immediately in front of the child was a computer screen. The children were instructed to control a computer mouse with their preferred hand.

The motor task setup was established using a customized software application (Matlab®, R2013b, Mathworks), allowing the child to control the vertical position (*y*-axis) of a cursor that moved left to right across the screen in 8 s i.e., with a constant speed. Based on the cursor’s automatic movement along *x*-axis and the child’s movement of the cursor on the *y*-axis, a trajectory was created, representing the trace drawn by the cursor’s movement during the respective trial.

Children were allowed 1 min of familiarization, during which they could freely move the cursor on an empty screen i.e., without a target. Following familiarization, actual motor practice (acquisition) was initiated. For each trial, the child was required to match the cursor as accurately as possible to a preset target (Roig et al., 2012). Eight different targets with different, but predictable trajectories were displayed in a random order (see Thomas et al., 2016b). All eight target trajectories started and ended at target mean positioned in the middle of the visual display, and all targets required the child to move the cursor both upwards and downwards from target mean.

For each trial, motor performance was calculated based on the root mean square error (RMSE) between the preset target and the trace produced by the child. In order to provide intuitive augmented feedback on motor performance to the children, motor performance was transformed to a 0–100 score. The score was defined as the mean of absolute vertical errors between the cursor and target in relation to target mean. If the error exceeded two times the distance from target to target mean, the score for this data point was set to zero. An RMSE of 0 equaled a score of 100 (see Thomas et al., 2016b).

The motor task was performed in blocks of 24 trials arranged so that each block contained three rounds of the eight different targets. Following each trial there was a 2 s pause during which augmented feedback on motor performance could be provided. To promote learning, the following feedback was provided: (1) knowledge of result (KR) was presented as the score ranging 0–100; (2) knowledge of performance was represented by a picture displaying the target with the trace produced by the child superimposed; (3) since the difficulty and thereby scores of individual traces varied, the child was provided with an average of KR for every full eight-targets cycle completed as a measure of average performance.

Augmented feedback was omitted from baseline and retention blocks to test memory and minimize the effect of feedback dependency and (re)learning (Salmoni et al., 1984). The children performed one block of baseline MT performance (A), followed by three blocks of acquisition (B_1_–B_3_). A 1-min break was allowed between adjoining blocks. Subsequently the children completed four blocks of MT to assess retention: immediately after (C), 1 h (D), 24 h (E) and 7 days (F) after motor practice, respectively (see Figure 1). One additional block of motor practice (block G) with augmented feedback was performed after the retention test on day seven to elucidate potential ceiling effects in motor performance.

#### Intervention Protocols

The intermittent exercise protocol used in both RUN and FLB was based on the exercise intervention applied in Roig et al. (2012). The intensity of the physical activity was relatively high, since some studies, including our own data (Thomas et al., 2016c), have indicated that high work intensity during consolidation leads to larger effect of the intervention (Angevaren et al., 2007; Winter et al., 2007).

Both FLB and RUN represent activities, which are frequently employed in school-settings. The activities were thus chosen based on ecological validity. While the physiological requirements of running in some aspects can be matched to those of FLB (e.g., average heart rate), FLB additionally involves more complex motor skills, decision making, teamwork and competition. The aim of the three group design was thus to include a passive control and if the results demonstrated differential effects of FLB and RUN, the design would suggest that differences would likely be related to the additional requirements of FLB in the aforementioned domains.

FLB played indoor FLB on a court measuring 6.6 × 14 m. The two teams each consisted of two children and one adult experimenter. The experimenters participated in the game to ensure that the flow and intensity of the game was maintained throughout the activity. The intensity of the game varied in a similar intermittent pattern for all children in FLB: 2 min instructions—in 3 min low intensity warm up—3 min of high intensity—2 min low intensity—3 min high intensity—2 min low intensity—3 min high intensity—2 min low intensity. Consequently the children performed a total of 9 min high intensity exercise. The running exercise took place at an indoor square track measuring 6.6 × 14 m. The children in RUN were required to exercise following an intermittent protocol as described for FLB thus totaling 9 min of high intensity running.

While performing high intensity exercise, the childrens’ heart rate was monitored online and results stored for offline analysis (Polar Team 2 System, Polar, Finland). This was also the case for three pilot experiments preceding the main experiment. The purpose of these pilot experiments was to determine the heart rates for children following the exercise protocol by playing FLB. During the main experiment, the aim was to reach similar heart rates as in the pilot experiments. This was ensured by experimenters monitoring heart rates online and verbally encouraging the children in both exercise groups. The children in the CON group were resting seated comfortably with the opportunity to watch cartoons for 20 min.

### Data Analysis

#### Children’s Characteristics and Exercise Data

Average heart rate (HR_avg_) and peak heart rate (HR_peak_) data were determined for each of the three intervals of high intensity exercise. Peak heart rate (HR_peak_) represented the highest heart rate observed during the exercise protocol and HR_avg_ the average of the peak heart rate observed in the three high intensity intervals. Differences in children’s characteristics between experimental groups were compared using one-way analysis of variance (ANOVA). A significant difference between groups was assumed if *p* < 0.05.

#### Motor Learning and Memory

Motor performance scores obtained in each 24-trial block were averaged, providing a total of nine data points for each child (block A to G). Motor learning and memory was assessed by measuring acquisition (block A to C) and retention (block C to G) separately. A one-way ANOVA was applied to investigate differences between groups at baseline (block A). This test was needed to rule out the possibility of differences in baseline motor skill performance. Motor learning was analyzed with two-way repeated measures (RM) ANOVA with TIME (block A to C) and GROUP as factors. Motor memory was analyzed with a two-way RM ANOVA with TIME (block C to G) and GROUP as factors.

In the second ANOVA model the motor performance scores in the delayed retention tests (i.e., retention at 1 h, 24 h and 7 days) were normalized to scores at immediate retention (block C). This was done to ensure that the analysis of motor memory factorized differences among groups in skill performance at the end of acquisition.

If sphericity was violated when applying an ANOVA (as determined using Mauchly’s test) the Greenhouse-Geisser correction was used. Furthermore, if a significant main or interaction effect was established in any ANOVA model, *post hoc* pairwise comparisons were performed using student’s *t*-test. To reduce the risk of type I errors, the α-level was adjusted to *p* ≤ 0.017 thus applying a modified Bonferroni’s correction procedure for the* post hoc* tests (0.05/3—0.05 divided by the number of comparisons within blocks; Roig et al., 2012). In addition, to elucidate if any statistical significant offline effects of memory could be documented within each experimental group, paired *t*-tests were carried out, comparing block C with blocks D, E, F and G, respectively. To reduce the risk of type I errors the α-level was adjusted to *p* ≤ 0.0125 (i.e., 0.05 divided by the number of comparisons within groups; Roig et al., 2012). To further investigate the time course of potential offline effects on motor skill retention, time-weighed regression analysis was performed within single subjects over the follow-up period including data from the immediate retention test and delayed retention at 24 h and 7 days (block C, E, F). A time-weighed slope measure was extracted for each individual, and entered into a one-way ANOVA to test for differences in offline mechanisms between groups. This procedure has previously been applied by Reis et al. (2009) to assess retention effects.

All statistical analyses were performed using IBM SPSS Statistics 22 for PC employing two-tailed probability tests. All *p*-values for *t*-tests are reported uncorrected. The results are provided as mean ± SEM unless otherwise reported.

## Results

### Description of Children and Groups

The baseline characteristics of the children participating in the three groups are summarized in Table 1. There were no significant differences between groups with regards to VO_2-peak_ (*F*_(2,69)_ = 1.33; *p* = 0.272), BMI (*F*_(2,74)_ = 1.56; *p* = 0.218), body fat percentage (*F*_(2,74)_ = 0.34; *p* = 0.717), PAQ_C_ score (*F*_(2,74)_ = 0.33; *p* = 0.743). There was a difference in age (*F*_(2,74)_ = 3.46; *p* = 0.037) with RUN being older compared to CON (*t* = 2.33; *p* = 0.024) and FLB (*t* = 2.02; *p* = 0.048). For heart rate measurements obtained during exercise, HR_peak_ was not different between exercise groups (*F*_(1,49)_ = 0.37; *p* = 0.374). In contrast, HR_avg_ was significantly higher during FLB compared to RUN (*t* = 2.86; *p* = 0.006).

### Motor Skill Acquisition

**Figure 2:**
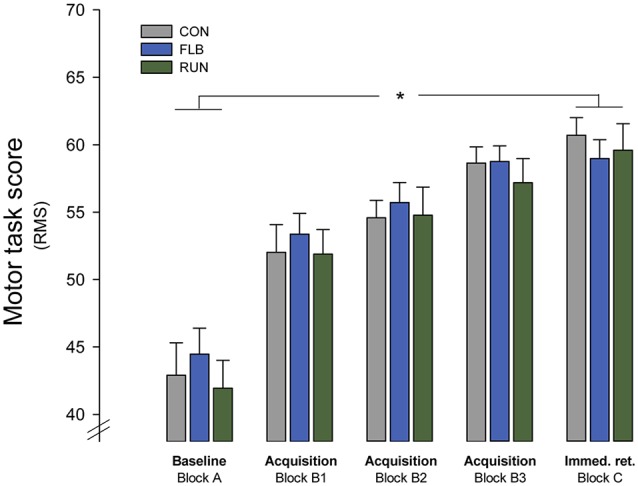
**Motor skill acquisition**. Group mean scores in the motor task at baseline (block A), during motor practice (blocks B1, B2, B3) and in the immediate retention test (block C; group mean ± SEM) (Immed. ret.: immediate retention test). *Significantly different (*p* < 0.05).

There were no differences in baseline motor performance between groups (*F*_(2,72)_ = 0.61; *p* = 0.549; see Figure 2). A two-way RM ANOVA assessing the effect of motor skill acquisition revealed no effect of either GROUP (*F*_(2,72)_ = 0.43; *p* = 0.654) or a GROUP-TIME interaction (*F*_(2,72)_ = 1.15; *p* = 0.322). Conversely there was an effect of TIME on motor performance baseline to immediate retention (*F*_(1,72)_ = 314; *p* < 0.001) across groups. These finding suggest similar baseline motor performance and skill acquisition for all groups (see Figure 2). The average motor performance score obtained at the immediate retention test following motor practice (59.61 ± 0.92) was significantly improved compared to average performance at baseline (43.67 ± 1.11; *t* = 18.42; *p* < 0.001).

### Delayed Retention

**Figure 3:**
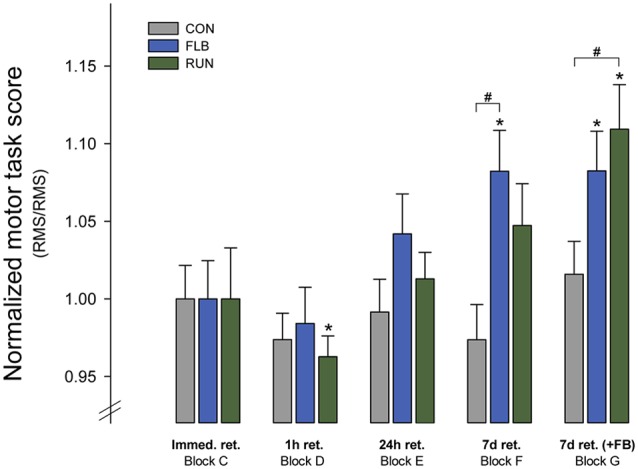
**Retention of motor skill**. Group mean scores in the motor task at block C to G for the three experimental groups (group mean ± SEM). The retention scores are normalized to performance in the immediate retention test (block C). Immed. ret.: immediate retention test; 1 h ret.: 1 h retention test; 24 h ret.: 24 h retention test; 7 d ret. −FB: 7 days retention without augmented feedback (KR); 7 d ret. +FB: 7 days retention with augmented feedback (KR); ^#^significantly different from control group (*p* < 0.017); *significantly different from immediate retention (*p* < 0.0125).

Motor performance scores obtained in delayed retention tests were normalized to immediate retention for the experimental groups to depict relative changes (Figure 3). Visual inspection of these results indicated that the exercise groups performed better in the delayed retention tests compared to CON, and the two-way RM ANOVA showed a statistically significant GROUP-TIME interaction (*F*_(5.972, 215)_ = 3.30; *p* = 0.004). In addition, there was a main effect of TIME on retention (*F*_(2.986, 215)_ = 15.83; *p* < 0.001), thus suggesting offline effects. *Post hoc* tests revealed that FLB performed significantly better than CON in block F (no augmented feedback on task performance) 7 days after motor practice (*t* = 3.12; *p* = 0.003). RUN also tended to perform better than CON (*t* = 2.09; *p* = 0.041, α–level adjusted *p* ≤ 0.017). There was no difference between FLB and RUN (*t* = 1.22; *p* = 0.228).

In block G however also 7 days after motor practice, the task was performed with augmented feedback on motor performance to investigate effects of continued practice. Within-block comparisons of motor performance for block G showed that RUN performed significantly better compared to CON, (*t* = 2.54; *p* = 0.014), and FLB still tended to perform better than CON (*t* = 1.97; *p* = 0.05, α–level adjusted *p* ≤ 0.017). There were no differences between FLB and RUN (*t* = 0.513; *p* = 0.611). The findings thus demonstrate that 7 days after initial motor practice, both FLB and RUN performed better compared to CON. Visual inspection of Figure 3 confirms that there was no ceiling effect for motor performance in the tracking task.

### Offline Effects within and between Intervention Groups

The assessment of offline effects in the three intervention groups revealed that children in RUN displayed a significant drop in motor performance from the immediate retention test (C) to block D, 1 h after the end of practice and 40 min after exercise (*t* = 2.76; *p* = 0.011). This decrease in motor performance was not observed for FLB or CON.

Assessment of offline effects across 24 h and 7 days showed that children in the FLB group displayed an offline gain in motor performance from the immediate retention test (block C) to block F (*t* = 3.11; *p* = 0.003) and block G (*t* = 3.23; *p* = 0.002) 7 days later. RUN also performed significantly better in block G after 7 days compared to block C (*t* = 4.0; *p* = 0.001), while there were no offline gains in motor performance for children in the CON group.

**Figure 4:**
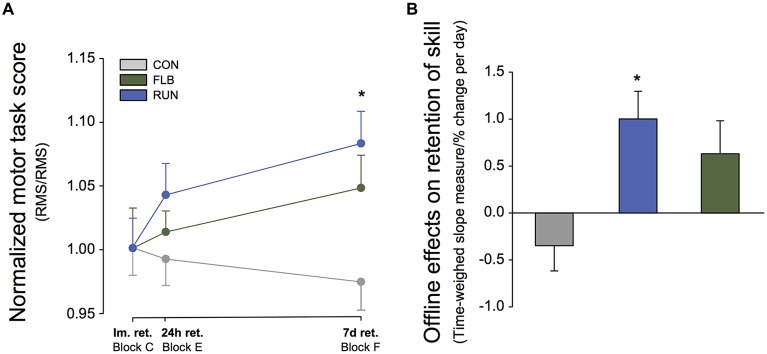
**Offline Effects. (A)** Changes in motor performance between the immediate retention test and the delayed retention tests without feedback (KR) at 24 h and 7 days (Block C, E, F). *Significantly different from control group (*p* < 0.017) and immediate retention (*p* < 0.0125). **(B)** Time-weighed slope measure for offline changes in motor performance between immediate retention, 24 h and 7 days retention (C,E,F), calculated within single subjects. *Significantly different from control group (*p* < 0.05).

Offline changes in motor skill were also assessed by means of time-weighed slope measure calculated within single subjects for changes in motor performance between the immediate retention test and delayed retention at 24 h and 7 days (blocks C, E, F; Figure 4). The one-way ANOVA revealed a significant effect of GROUP (*F*_(2,75)_ = 5.0; *p* = 0.009) and *post hoc* comparisons revealed that the slope parameter was significantly higher in FLB (*t* = 3.32; *p* = 0.0017) compared to CON (Figure 4B). The slope parameter also tended to be higher for RUN compared to CON (*t* = 2.23; *p* = 0.032, α–level adjusted *p* ≤ 0.017). There was no difference between FLB and RUN (*t* = 0.863; *p* = 0.39). These findings indicate offline gains in motor memory for both exercise groups compared to the control group.

## Discussion

The present study is to our knowledge the first to investigate: (1) whether a single bout of acute intense intermittent exercise following skill acquisition can improve motor memory consolidation in preadolescent children; and (2) whether different types of acute exercise employed in a school setting are accompanied by differential effects on motor memory and skill learning.

The results demonstrate that an acute bout of intense exercise performed after practicing a novel motor task improves long-term motor memory. In accordance with Roig et al. (2012) we have thus demonstrated that exercise can facilitate retention following skill learning through an effect on motor memory consolidation also in pre-adolescent children. Furthermore, we investigated the effects of different exercise interventions including running and FLB, which represents a team-oriented exercise intervention, demonstrating that team-sports can be effective in reinforcing the consolidation and retention of long-term motor memory. The finding that long-term motor memory was enhanced in both exercise groups indicates that the type of exercise does not seem to be essential to achieve a memory-facilitating effect. Most likely, timing, intensity and duration of exercise are more important parameters for facilitating memory consolidation processes (Angevaren et al., 2007; Chang et al., 2012; Roig et al., 2013; Thomas et al., 2016a,c).

While no study to our knowledge assessed effects of acute exercise on motor memory consolidation in preadolescents, Pesce et al. (2009) previously demonstrated a positive effect of acute exercise on declarative memory in this group, when submaximal exercise preceded the memory encoding and retrieval. In this case, exercise may have influenced both declarative memory formation and storage. Furthermore, Pesce et al. (2009) did not assess long-term retention thereby potentially missing further offline effects of the exercise. Delayed retention tests are highly important in order to assess interventional effects on consolidation (Kantak and Winstein, 2012), since the formation of both long-term declarative (McGaugh, 2000) and motor memory as in the present study (Brashers-Krug et al., 1996) can persist for many hours after acquisition. In the present study, skill acquisition preceded exercise, and this design including delayed retention tests enable us to demonstrate that the acute bout of exercise, enhanced memory through an effect on consolidation processes.

The timing of both the exercise relative to skill acquisition and the delayed retention tests are important in order to elucidate the effects of exercise on motor memory (Statton et al., 2015; Roig et al., 2016; Thomas et al., 2016a). In the current study, exercise followed motor practice with a short delay, which may be important for the positive effect on consolidation (Roig et al., 2016; Thomas et al., 2016a). Indeed, Thomas et al. (2016a) recently demonstrated that the temporal proximity between the exercise bout and motor practice has a positive influence on motor memory consolidation. This may relate to the potential temporal gradient of the consolidation processes following motor practice.

It is noteworthy that significant effects of exercise on motor memory improvements may appear long after the performance of the exercise bout and not immediately after. Indeed in the present study, memory and thus motor performance was significantly enhanced in the exercise groups 7 days after encoding, even though there was a detrimental effect in the running group 1 h after motor practice. Other studies have found non-significant or even detrimental effects of acute exercise on memory when retention tests were employed during or shortly after a moderate to high intensity exercise bout (Roig et al., 2013). This approach is not appropriate to assess effects on memory because the proximity of the retention test to the exercise stimulus can easily mask potential gains in memory due to exercise-induced fatigue and/or arousal, particularly when exercise is performed at a high intensity (Roig et al., 2016). Thus although intense running was accompanied by an acute detrimental effect on motor performance, this did not preclude delayed gains in the RUN group.

The finding of improved motor memory for the exercise groups 7 days after motor practice is consistent with previous studies (Roig et al., 2012; Skriver et al., 2014; Thomas et al., 2016a,c). While previous studies have also found significant effects of exercise 24 h after motor practice, this was only a tendency for FLB in the current study. Both groups did however display offline improvements from immediate retention to the 7-day retention test (see Figures 3, 4). It is possible that the effects of exercise evolve long after synaptic consolidation processes (Dudai, 2012) and that several days are required to see the effects of exercise on memory consolidation. Sleep-dependent processes may also be involved in the exercise-induced improvements in motor memory consolidation and this could contribute to the delayed effects (Dudai, 2012). In addition, the retrieval and motor practice inherent in the 24 h retention test and the following reconsolidation may contribute to the effects on delayed motor memory observed at 7 days.

Although there were no significant differences between the two exercise groups and both groups displayed different degrees of offline gains in motor performance, it is also noteworthy that there seemed to be apparent differential effects. Whereas FLB demonstrated significant offline improvements from immediate retention to 7 days retention and performed better compared to the control group, this effect was less pronounced for RUN. The running group however displayed a marked gain in performance with continued motor practice on day 7. In line with this, Rhee et al. (2016) also recently noted a latent effect of exercise on the development of motor performance with continued practice, and this may represent an alternative way in which exercise-mediated consolidation effects can influence motor memory and development of motor skills. Thus, acute exercise may both promote both consolidation processes, reconsolidation and facilitate learning with continued motor practice differentially. In essence however, both FLB and RUN ultimately led to improvements in motor memory in the current study.

Moderate to vigorous acute exercise preceding encoding has previously been demonstrated to promote declarative memory in children (Pesce et al., 2009) and adults (Winter et al., 2007) as well as motor skill acquisition (Statton et al., 2015). In a recent study by Thomas et al. (2016c) it was found that higher intensity exercise following skill acquisition was accompanied by more pronounced effects on motor memory compared to moderate intensity exercise. In agreement with previous studies in adults (Roig et al., 2012; Skriver et al., 2014) the present results demonstrate that high-intensity exercise following skill learning can promote consolidation of memory in children.

Indeed children in both exercise groups displayed high heart rates and thus performed exercise at a high intensity during the intermittent intervention. The groups displayed small differences concerning age and heart rate during exercise. The difference in age for RUN occurred due to an unexpected delay due to national labor disputes, causing a relatively larger part of children in RUN to participate in the main experiment 2 months later than planned. Since the groups were matched prior to initiation of the main experiment, this led to the observed small but significant difference for RUN. However, since neither age nor HR_avg_ were related to acquisition (age: *r* = −0.111; *p* = 0.338; HR_avg_: *r* = 0.171; *p* = 0.229) or retention (age: *r* = 0.109; *p* = 0.348; HR_avg_: *r* = 0.003; *p* = 0.99) these differences were considered to be of minor importance to the interpretation and validity of the results.

While mean HR was high and comparable between exercise groups, FLB and running are naturally different in several aspects. While running is a continuous activity, FLB naturally is a more intermittent activity and although exercise-protocols were also matched on time, the inherent intermittent nature of FLB may have influenced the anaerobic metabolism compared to running. FLB also involves decision making, varied movements also for the upper body in addition to a team element and competition. It is based on the design of the present study not possible to say to which extent these differences may have influenced the results, but further studies may elucidate whether these factors could influence motor memory processes.

The finding in the present study that motor memory consolidation and long-term motor memory can be enhanced by both FLB and running is consistent with the findings of Thomas et al. (2016b) in adults. This does however not mean that it is not relevant to consider exercise type. The finding that team sport in addition to running can boost long-term memory in children is important, since the classic experimental exercise regimes on treadmills or bike ergometers, are far from the regular activities that pre-adolescent children experience in school settings, e.g., playing during recess or PE. The applicability of team sports allows flexibility in selecting a more motivating and manageable activity depending on individual and group preferences.

A recent study has documented that the fitness levels of the younger population are declining (Tomkinson and Olds, 2007) making any effort that stimulates participation in regular physical activity ever more relevant. Furthermore, the level of fundamental movement skills in childhood is correlated to level of physical activity and obesity (Morgan et al., 2013) and physical activity can mediate the association between childhood motor functions and adolescents’ academic achievements (Kantomaa et al., 2013). Since increased focus on motor skills combined with incorporation of exercise in education institutions can benefit both skill learning and academic achievements as demonstrated by Ericsson and Karlsson (2014), this underlines the importance of early-life motor skill training.

Skill learning is important and the mechanisms subserving formation and retention of declarative and nondeclarative memory (Censor et al., 2012) are to a large degree similar. Although memory systems are distinct, recent studies have made it conceivable that declarative and procedural memories can and do indeed interact (Robertson, 2012), and memories are not necessarily confined to independent systems. The functional connection between memory processes may contribute to the observed long-term effects of motor skill training on academic performance (Ericsson and Karlsson, 2014), and it suggests that consolidation of declarative memory might also benefit from acute exercise (Kandel et al., 2014). Caution should however be taken when extrapolating the current results to other types of memory or exercise, and studies assessing the long-term effects of acute exercise on declarative and nondeclarative memory are needed to conclusively document such effects.

The present results demonstrate that acute exercise in temporal proximity to a skill learning session can facilitate memory consolidation following acquisition and thus retention of motor memory. Based on the obtained measurements, it is difficult to infer in detail, which mechanisms could be involved in the observed effects of exercise on motor memory. Given that exercise was placed immediately following motor practice, long-term motor memory is most likely influenced by positive influences of the exercise on consolidation processes involving neuroplastic changes in the central nervous system (Brashers-Krug et al., 1996; Lundbye-Jensen et al., 2011; Taubert et al., 2015). We have in a previous study in adults found relations between exercise-induced effects on motor memory and changes in concentrations of specific biomarkers in peripheral blood samples (Skriver et al., 2014). This analysis demonstrated that higher concentrations of brain derived neurotrophic factor (BDNF), norepinephrine and lactate correlated with better retention of motor memory. Recently, a similar relationship was found between delayed motor memory and changes in corticospinal excitability following exercise (Ostadan et al., 2016). Whether these associations may also be found for preadolescent children remains to be elucidated. Future studies could thus focus on investigating the mechanisms underlying the observed effects through application of e.g., electrophysiological or neuroimaging techniques in children.

In addition to investigating the mechanisms underlying the behavioral effects observed in the current study, future studies could investigate the potential effects of strategically implementing acute exercise in longer interventions thus combining acute and chronic exercise with the aim of influencing e.g., skill learning and motor memory. In addition to the direct transferability of the behavioral findings to school settings, the results of the present study could also be relevant for rehabilitation training with the purpose of improving long-term outcome of the rehabilitative treatment (Vaynman and Gomez-Pinilla, 2005), with acquisition and retention of motor skills being particularly important for physical rehabilitation. However, studies targeting clinical populations with mobility impairments are required before finally making such recommendations.

## Conclusion

The present study is to our knowledge the first to demonstrate that acute intense intermittent exercise performed immediately after motor skill acquisition facilitates long-term motor memory in pre-adolescent children, presumably by promoting memory consolidation. The results also demonstrate that the effects can be accomplished in a school setting. The positive effect of exercise both as a team game (i.e., FLB) and running indicates that the observed memory improvements are determined to a larger extent by physiological factors such as intensity and timing of exercise, rather than the types of movements performed.

## Author Contributions

JL-J, KS, JBN and MR designed the experiment. JL-J and KS collected the data. JL-J, KS and MR conducted the required data analysis. All authors contributed to drafting the manuscript, and approved the final version of the manuscript.

## Funding

The study was supported by The Ludvig & Sara Elsass Foundation and Nordea-fonden.

## Conflict of Interest Statement

The authors declare that the research was conducted in the absence of any commercial or financial relationships that could be construed as a potential conflict of interest.
